# Phylogenetic analysis of *Trichostrongylus vitrinus* isolates from southwest Iran

**DOI:** 10.1186/s13071-020-04438-y

**Published:** 2020-11-07

**Authors:** Mohammad Amin Ghatee, Seyed Ali Asghar Malek Hosseini, Masoud Marashifard, Mehdi Karamian, Walter Robert Taylor, Ali Jamshidi, Iraj Mobedi, Hasan Azarmehr

**Affiliations:** 1 Cellular and Molecular Research Center, Yasuj University of Medical Sciences, Yasuj, Iran; 2 Department of Parasitology, School of Medicine, Yasuj University of Medical Sciences, Yasuj, Iran; 3 Student Research Committee, Yasuj University of Medical Sciences, Yasuj, Iran; 4 Department of Parasitology and Mycology, School of Medicine, Zanjan University of Medical Sciences, Zanjan, Iran; 5 Mahidol Oxford Tropical Medicine Research Unit, Bangkok, Thailand; 6 Oxford Centre for Tropical Medicine and Global Health, University of Oxford, Oxford, UK; 7 Department of Medical Parasitology and Mycology, School of Public Health, Tehran University of Medical Sciences, Tehran, Iran

**Keywords:** *Trichostrongylus vitrinus*, Phylogenetic analysis, ITS2-rDNA

## Abstract

**Background:**

*Trichostrongylus* is one of the most important zoonotic trichostrongylid nematodes, infecting mostly livestock. Data on its genetic characteristics are lacking in Iran.

**Methods:**

We determined the phylogenetic relationships of *Trichostrongylus* species in three counties of Kohgiloyeh and Boyerahmad (K-B) province, southwest Iran. Small intestine and abomasum of 70 sheep and goats were investigated.

**Results:**

A total of 35 isolates of *Trichostrongylus* worms were detected and all were genetically identified as *Trichostrongylus vitrinus*. Analysis of 321 bp of the internal transcribed spacer 2 (ITS2) of ribosomal DNA revealed 16 genotypes. All genotypes were single nucleotide polymorphisms, including some hypervariable points. All sequences were trimmed to 170 bp, compared with sequences on GenBank including short sequences from other endemic foci of Iran and other countries and all isolates were used to generate a maximum likelihood phylogenetic tree, which consisted of two clades A and B. Clade A included isolates from Iran, Russia, New Zealand, Australia and the UK; clade B only contained South African isolates. Most clade A isolates (north, southwest and west Iran, Russia, New Zealand, Australia and UK) were in a similar phylogenetic position. One subclade was detected in clade A (isolates from Southwest Iran, New Zealand and UK).

**Conclusions:**

We hypothesize that drug resistant *T. vitrinus* may account for its exclusive detection in our samples. The high similarity of genotypes from Iran, New Zealand and UK may be due to their close political relationships during the colonial era. More research is needed to understand better the phylogeny of *T. vitrinus* and its relationship with drug resistance and human transmission. 
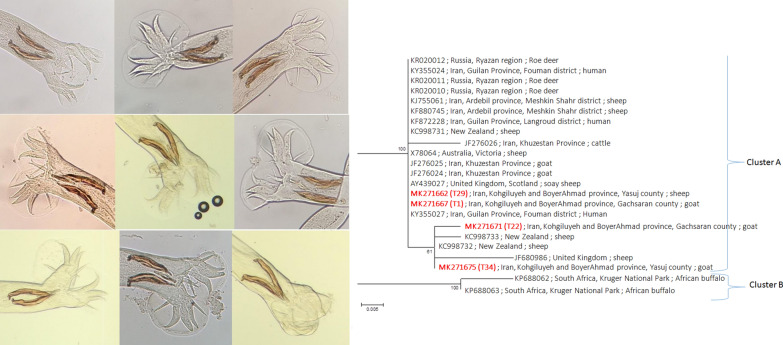

## Background

Parasitic infections affect populations all over the world, especially in developing countries. Among the gastrointestinal nematodes, *Trichostrongylus* (order Strongylida, family Trichostrongylidae), has a substantial economic, medical and veterinary impact. *Trichostrongylus* spp. comprise more than ten principal zoonotic species, that affect human and livestock such as goats, sheep and cattle and cause gastroenteritis, diarrhea, weight loss and losses of production in livestock [1] and a spectrum of manifestations from subclinical symptoms to rash, abdominal pain, diarrhea, mild anemia, leukocytosis and eosinophilia in humans [2]. Moreover, anthelmintic resistance of nematodes in this genus is common to drugs like pyrantel pamoate, levamizole and albendazole and results in treatment failure and increased livestock morbidity [3, 4].

Trichostrongylosis in livestock has been reported from diverse geographical settings, including Southeast Asia [5], the Middle East [6], Africa [7], Europe [8, 9] and Australian continent [4]. Similarly, human infection has been reported from Italy [10], France [11], Morocco and Egypt [12, 13], Brazil [14], the Caribbean [15] and Australia [16]. Iran is one of the main foci of livestock and human infections and trichostrongylosis has been reported frequently [17, 18].

The life-cycle of *Trichostrongylus* spp. is direct, simple and without an intermediate host. Host infection occurs upon ingestion of filariform larvae found in contaminated vegetables or water. These larvae migrate to the small intestine or abomasum, grow to adults which mate and produce eggs that are excreted in the feces into the external environment. Under optimal temperature and humidity, rhabditiform larvae hatch from eggs within a few days and, after two moltings (L1 and L2) over 5 to 10 days, become infective filariform larvae (third-stage) [3, 19].

Clinical diagnosis of infection is based on the finding of eggs in the feces of human and herbivores but the majority of eggs of species of the family Trichostrongylidae, except for the genus *Nematodirus*, are indistinguishable from each other. Moreover, *Trichostrongylus* spp. eggs may be confused with hookworm eggs. Although traditional morphological and morphometric methods can identify the adult male worms from different *Trichostrongylus* species, they are time-consuming and laborious and cannot be used for the species identification of ova and female worms [20].

With the advent of molecular techniques in recent years, many problems in identification have been resolved. Several DNA based techniques can discriminate different species of *Trichostrongylus*, including all life-cycle stages and sexes, e.g. PCR-restriction fragment length polymorphism (PCR-RFLP) [21], randomized amplified polymorphism DNA (RAPD) [22], PCR-single-strand conformational polymorphism (PCR-SSCP) [23], PCR-denaturing gradient gel electrophoresis (PCR-DGGE) [24], multiplex-PCR [25], quantitative PCR [26] and sequence analysis [27].

RAPD-PCR, PCR-SSCP and PCR-sequence analysis have been used to study the phylogenetic relationships and population structure of strongylid nematodes [28] and gene sequencing has led to the development of genetic databases for inter- and intraspecific identification [29].

The internal transcribed spacer 2 region of the ribosomal RNA gene (ITS2-rDNA) is a useful marker to detect interspecific [30–32] and intraspecific differences [32–34] and establish the phylogenetic relationships within the family Trichostrongylidae.

There are few data on the genetics of *Trichostrongylus* species in Iran. We applied sequence analysis of ITS2 to conduct a study with the aim of investigation of phylogenetic relationships of *Trichostrongylus* isolates in southwest Iran.

## Methods

### Study area

This study was conducted in the three main counties of Kohgiloyeh and Boyerahmad (K-B) Province: Boyerahmad, Gachsaran and Kohgiloyeh. This southwestern province is mostly mountainous with 20% of the region comprising plains. The highest peak and the plains are 4283 and 115 m above sea level, respectively. The difference in elevation in the northeast (Boyerahmad county) and the south-southwest (Gachsaran and Kohgiloyeh counties) results in two different types of weather patterns; Boyerahmad county is cold and wet and covered with large oak forests while Gachsaran and Kohgiloyeh are warm and semi-arid regions.

Although most of people live in rural and urban areas in K-B, this province also has a significant nomadic population. Every year, the nomads herd their sheep and goats to and from their summer (Yailaq) and winter quarters (Qishlag). Data from the Veterinary Bureau consensus report that the number of sheep, goats and cattle in K-B province is 1,543,300.

### Sample collection

Samples were obtained from a total of 70 slaughtered sheep and goats from the three counties. Their small intestines and abomasa were separated from the carcasses in the slaughterhouses, separately packed in plastic bags, and sent to the Parasitology laboratory of Yasuj University of Medical Science. Mucosa were scraped and the contents of the small intestines and abomasa were washed separately onto a 100-mesh sieve (aperture size 0.149 mm). Washed contents and mucosal scrapings were examined under a stereo microscope to facilitate the recovery of worms. Samples were stored in 70% ethanol until molecular analysis.

The identification of *Trichostrongylus* spp. was carried out based on morphological characteristics. Each adult male worm was identified by its morphological and morphometric characteristics and then subjected to sequence analysis of the ITS2 region for further identification [3].

### DNA extraction

300 μl of lysis buffer (NaCl 0.1M, EDTA 0.01M, Tris-HCL 0.1M, SDS 1%) was added to the microtube containing the *Trichostrongylus* worm and DNA extraction performed in two steps. Step one used the freezing-thawing technique to disrupt the worm tegument and cells. Samples were frozen in liquid nitrogen (1–2 min) and thawed at room temperature with extra crushing using a steel bullet that was added to each microtube (20–30 s). In the second step, 30 µg/ml of proteinase K was added and the samples were incubated at 56 °C for 1 h; then the DNA was extracted once with phenol/chloroform (25:24 v/v) and again with chloroform. DNA was precipitated with equal volumes of isopropanol and also one tenth volume of 3 M NaAc. The DNA precipitate was washed with 70% ethanol, dried, and dissolved in 50 µl deionized water, and stored at – 20 °C.

### ITS2 PCR and sequencing

Primers NC1 (5'-ACG TCT GGT TCA GGG TTG TT-3') and NC2 (5'-TTA GTT TCT TTT CCT CCG CT-3') [35] were used to amplify the ITS2 region of all samples and confirm the genus *Trichostrongylus*. The PCR reactions were performed in a final volume of 25 μl containing 12.5 μl of 2× premix (Ampliqon, Skovlunde, Denmark), 20 pmol of each primer, 5 μl of template DNA, and enough water made up to 25 μl. Optimal conditions for PCR were initial denaturation at 95 °C (7 min) followed by 35 cycles of 95 °C (45 s), 56 °C (45 s), and 72 °C (50 s), and a final extension step at 72 °C (5 min). The PCR products were visualized after electrophoresis on a 1.5% agarose gel with 0.5 μg/ml ethidium bromide. A 100 bp DNA marker was used for sizing the bands in each run.

The PCR products were also sent to Bioneer Company (Daejeon, South Korea) and sequenced using the Applied Biosystems automated DNA sequencer (3730 XL; Applied Biosystems, Foster City, USA). Sequencing was performed in both directions using the same PCR primers. The sequences were deposited in the GenBank database under accession numbers MK271662-MK271677.

### Phylogenetic analysis

**Fig. 1:**
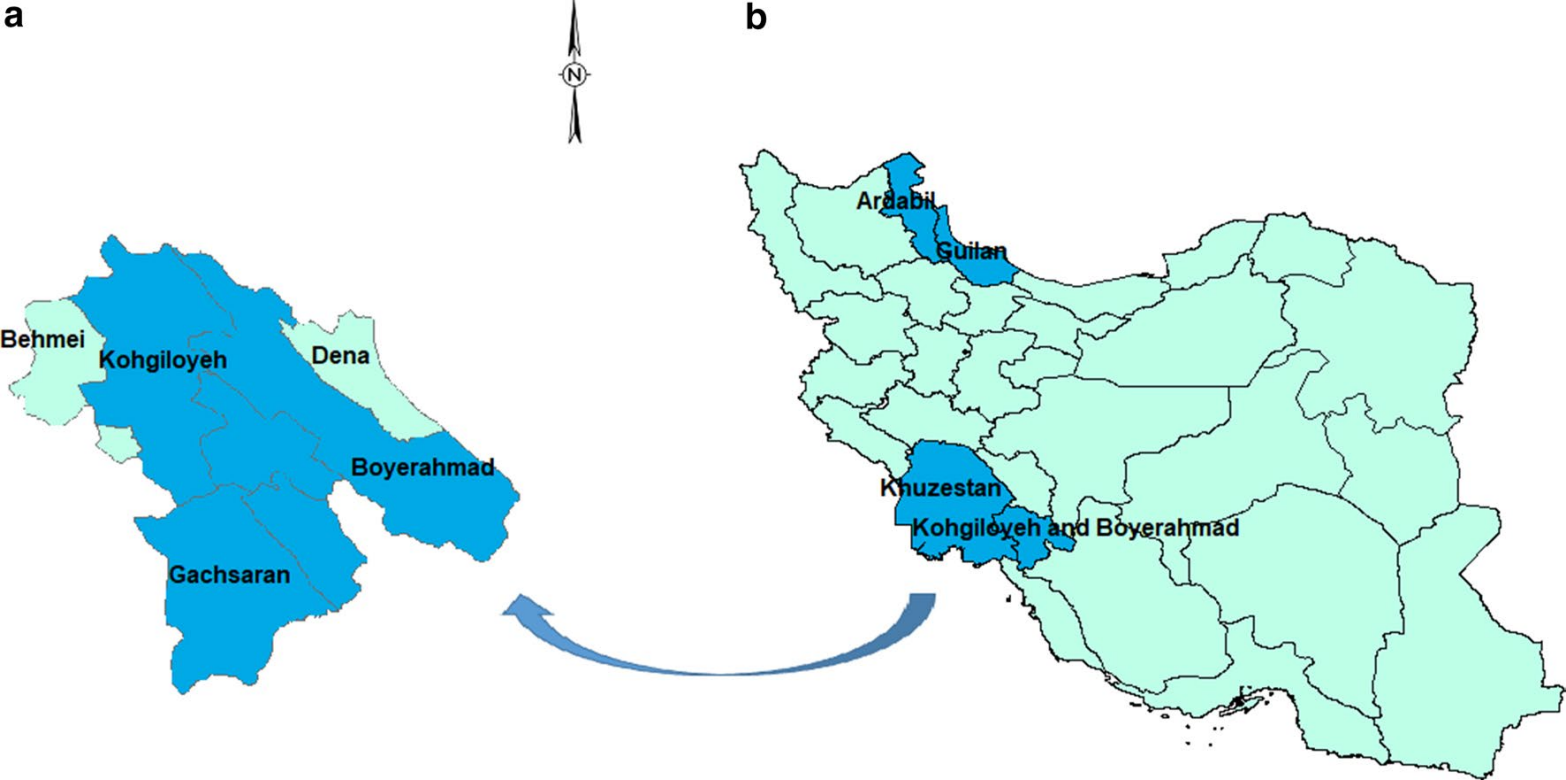
Maps showing Kohgiloyeh and Boyerahmad Province counties (**a**) and the Iranian provinces (**b**) from which *T. vitrinus* isolates were used for generating the phylogenetic trees. The geographical origins of *T. vitrinus* are shown in dark blue

BLAST software (https://www.ncbi.nlm.nih.gov) was used to compare the newly generated sequences with published ITS2 sequences in GenBank to identify and reconfirm species identification based on morphological and morphometric data. The sequences were analyzed using Geneious Pro 5.5.6 software. Two phylogenetic trees were generated using: (i) all our isolates; and (ii) including our genotypes (STs) and all other *T. vitrinus* ITS2 sequences from other regions of Iran and the other countries that were deposited in GenBank (Fig. 1). Alignment was performed using ClustalW and nucleotide distances were calculated by the BioEdit software (version 7.0.5.3) [36]. Maximum likelihood trees were inferred by using MEGA 6 software [37] after trimming all sequences at both ends. Bootstrap values for the ML method were based on 1000 replicates.

### Results

Overall, 35 *Trichostrongylus* isolates were obtained from more than 2000 nematodes in the abomasum and small intestines: (i) 8 from Boyerahmad county (sample ID: T4 and T29 to T35); (ii) 10 from Kohgiloyeh county (T5 to T14); and (iii) 17 from Gachsaran county (T1 to T3 and T15 to T28) (Table 1). *Trichostrongylus vitrinus* was the only species identified based on the morphological and morphometric characteristics. Amplification of the ITS2 fragment resulted in bands of about 321 bp for all 35 isolates and identified 16 different genotypes. The highest number of isolates belonged to genotypes 1 (*n* = 7), 6 (*n* = 4), 7 (*n* = 4), 5 (*n* = 3) and 10 (*n* = 3). Genotypes 1, 2 and 6 were found in all three counties, while genotypes 7 and 10 were observed only in Kohgiluyeh/Gachsaran and Boyerahmad/Gachsaran, respectively. Genotypes 3 and 5 were each seen in two isolates and genotypes 9 to 16 each contained only one isolate (Table 2).

**Table 1:** The sex, host type, isolation site and geographical origin of *T. vitrinus* isolates in this study

Sample ID	Sex	Host	Isolation site	Geographical origin (county)
T1	Male	Goat	Small intestine	Gachsaran
T2	Female	Goat	Small intestine	Gachsaran
T3	Male	Goat	Small intestine	Gachsaran
T4	Female	Sheep	Abomasum	Boyerahmad
T5	Male	Sheep	Abomasum	Kohgiluyeh
T6	Male	Sheep	Abomasum	Kohgiluyeh
T7	Male	Sheep	Abomasum	Kohgiluyeh
T8	Male	Sheep	Abomasum	Kohgiluyeh
T9	Male	Sheep	Abomasum	Kohgiluyeh
T10	Male	Sheep	Abomasum	Kohgiluyeh
T11	Male	Sheep	Abomasum	Kohgiluyeh
T12	Male	Sheep	Abomasum	Kohgiluyeh
T13	Male	Sheep	Small intestine	Kohgiluyeh
T14	Male	Sheep	Abomasum	Kohgiluyeh
T15	Female	Goat	Small intestine	Gachsaran
T16	Male	Goat	Small intestine	Gachsaran
T17	Male	Goat	Small intestine	Gachsaran
T18	Male	Goat	Small intestine	Gachsaran
T19	Male	Goat	Small intestine	Gachsaran
T20	Male	Goat	Small intestine	Gachsaran
T21	Male	Goat	Small intestine	Gachsaran
T22	Male	Goat	Small intestine	Gachsaran
T23	Male	Goat	Small intestine	Gachsaran
T24	Male	Goat	Small intestine	Gachsaran
T25	Male	Goat	Small intestine	Gachsaran
T26	Male	Goat	Small intestine	Gachsaran
T27	Male	Goat	Small intestine	Gachsaran
T28	Male	Goat	Small intestine	Gachsaran
T29	Male	Sheep	Abomasum	Boyerahmad
T30	Male	Sheep	Abomasum	Boyerahmad
T31	Male	Goat	Abomasum	Boyerahmad
T32	Male	Goat	Abomasum	Boyerahmad
T33	Male	Goat	Abomasum	Boyerahmad
T34	Male	Goat	Abomasum	Boyerahmad
T35	Male	Goat	Abomasum	Boyerahmad

**Table 2:** Single nucleotide polymorphism positions of 16 genotypes and 4 haplotypes among long (321 bp) and short (170 bp) ITS2 sequences, respectively, from *T. vitrinus* isolates in southwest Iran

Haplotype (170 bp)	SNPs	Sample ID	SNPs	Genotype and GenBank ID (321 bp)
Haplotype A	85T > C	T1; T2; T3; T4; T17; T18; T28	147T > C	Genotype 1 (MK271667)
		T9; T24; T35	37C > A; 147T > C	Genotype 2 (MK271664)
		T6; T16	62C > T; 147T > C	Genotype 3 (MK271663)
		T7; T21	37C > A; 62C > T; 147T > C	Genotype 4 (MK271666)
		T15; T20	51T > C; 147T > C	Genotype 5 (MK271674)
Haplotype B		T8; T25; T29; T31		Genotype 6 (MK271662)
		T10; T14; T19; T23	51T > C	Genotype 7 (MK271665)
		T13	47_48insT; 51T > C	Genotype 8 (MK271673)
		T5	37C > A	Genotype 9 (MK271669)
Haplotype C	34T > A; 85T > C	T26; T33; T34	62C > T; 96T > A; 147T > C	Genotype 10 (MK271675)
		T11	47_48insT; 51T > C; 96T > A; 147T > C	Genotype 11 (MK271672)
		T27	51T > C; 96T > A; 147T > C	Genotype 12 (MK271676)
		T32	96T > A; 147T > C	Genotype 13 (MK271677)
		T12	37C > A; 96T > A; 147T > C; 246T > C	Genotype 14 (MK271670)
		T30	37C > A; 62C > T; 96T > A; 147T > C	Genotype 15 (MK271668)
Haplotype D	34T > A; 85T > C; 147A > G	T22	37C > A; 62C > T; 96T > A; 147T > C; 209A > G; 246T > C	Genotype 16 (MK271671)

Most of the nucleotide polymorphisms were observed in nucleotides that were close to the 5'-end of the sequences and all were single nucleotide polymorphisms (SNP). T to C transition at nucleotide 147 was the most frequent SNP observed in all genotypes except genotypes 6–9. Additional transitions found were T to C at nucleotides 51 (genotypes 5, 7, 8, 11–12) and 246 (genotypes 14 and 16), C to T at nucleotides 62 (genotypes 3–4, 10, 15–16), A to G at nucleotide 209 (genotype 16). T to A transversion at nucleotide 96 (genotypes 10–16) and C to A transversion at nucleotide 37 (genotypes 2, 4, 9 and 14–16) were also observed as well as insertion of A at nucleotide 48 (genotypes 8 and 11) (Table 2).

**Fig. 2:**
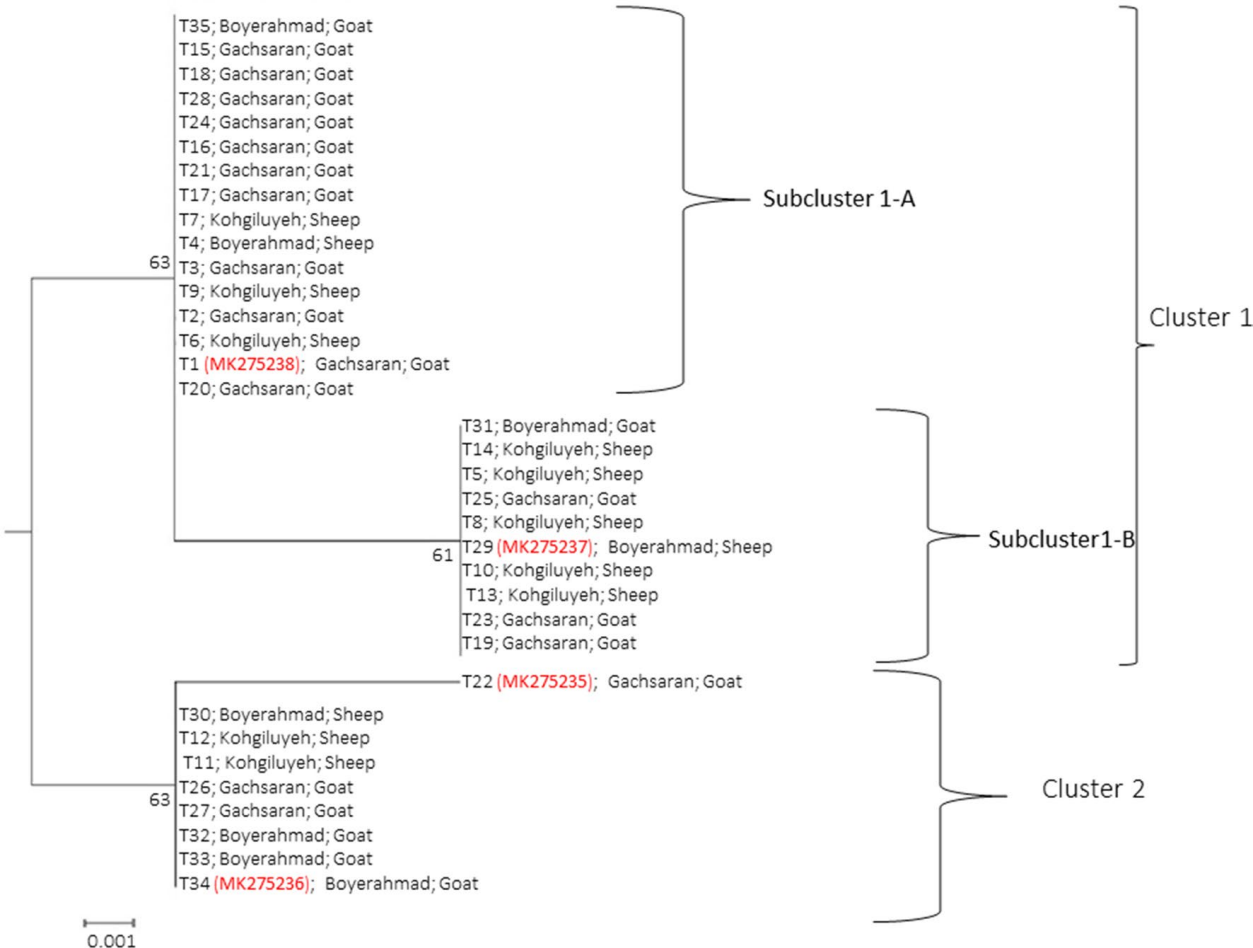
Maximum likelihood phylogenetic tree based on the Tamura-Nei model constructed from ITS2 sequences of *T. vitrinus* from Kohgiloyeh and Boyerahmad Province in southwest Iran. Geographical origin (county) and host for each isolate is shown. Representative sequences generated in the present study are indicated in red

Because there was a number of short STs in the GenBank, all STs from our study, other parts of Iran and other countries were trimmed and shortened after alignment and eventually a 170 nucleotide fragment of the sequences was obtained for the phylogenetic analysis (Fig. 2). Based on the polymorphisms in this short fragment, only 4 haplotypes were defined for our K-B isolates (named A to D). Haplotypes A, B, and C were observed in Boyerahmad, Gachsaran and Kohgiloyeh but Haplotype D was reported only from Gachsaran. Haplotypes B and D had the greatest genetic distance (0.018%) (Tables 2, 3).

**Table 3:** Genetic distances as calculated by BioEdit (7.05.8) between haplotypes A, B, C and D of isolates in this study

Haplotype	A	B	C	D
A				
B	0.006			
C	0.006	0.012		
D	0.012	0.018	0.006	

**Fig. 3:**
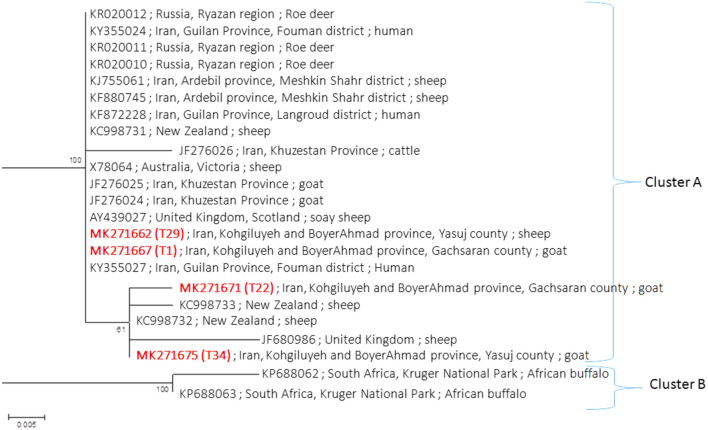
Maximum likelihood phylogenetic tree of *T. vitrinus* from Iran and other countries

The constructed phylogenetic tree of our 35 *T. vitrinus* isolates (Fig. 2) revealed two main clades, 1 and 2. Clade 1 contains two subclades 1-A and 1-B. Subclade 1-A consisted of all isolates of haplotype A, including: 11 isolates from Gachsaran; 3 from Kohgiluyeh; and 2 from Boyerahmad. Subclade 1-B included 4 isolates from Gachsaran, 5 from Kohgiluyeh and 1 isolate from Boyerahmad; all from haplotype B. Clade 2 consisted of 9 isolates from haplotypes C and D. Eight isolates (4 from Boyerahmad, 2 each from Gachsaran and Kohgiluyeh) showed haplotype C. The haplotype D isolate from Gachsaran was in a more distant phylogenetic position. Both main clades included isolates from both hosts and both small intestine and abomasum. The second phylogenetic tree consisted of representatives of haplotypes of K-B province (A to D haplotypes) and all reliable ITS2 STs from other regions of Iran and other countries that retrieved from GenBank. Haplotypes distribution by region is shown is Table 4. Based on the tree topology, there were two clades, A and B (Fig. 3). Clade A showed little resolution and contained more genotypes from a broader geographical reach encompassing north (KY355024, KY355027 and KF872228), northwest (KJ755061 and KF880745) west (JF276024, JF276025 and JF276026) and K-B province in southwest (MK271662, MK271667, MK271671 and MK271675) Iran, the Ryazan region from Russia (KR020010, KR020011 and KR020012), the UK (AY439027 and JF680986), Australia (X78064) and New Zealand (NZ) (KC998731, KC998732 and KC998733), while Clade B isolates were all from one national park in South Africa (KP688062 and KP688063). Most clade A genotypes had similar phylogenetic positions but some NZ (KC998732 and KC998733) and the UK (JF680986) isolates and haplotypes C and D from Kohgiloyeh and Boyerahmad Province (MK271671 and MK271675) consisted one subclade in this clade, though it was not well supported by bootstrap analysis. Also, one isolate from west Iran (JF276026) was placed in the main part of clade A.

**Table 4:** *Trichostrongylus vitrinus* ITS2 genotypes (STs) from the present study, other parts of Iran and other countries retrieved from GenBank used to generate the phylogenetic tree

Host	Location	GenBank ID	Reference
Soay sheep	UK (Scotland)	AY439027	Wimmer et al. [49]
Sheep	New Zealand	KC998731-KC998733	Bisset et al. [48]
African buffalo	South Africa: Kruger National Park	KP688062; KP688063	Budischak et al. [52]
Sheep	Australia: Victoria	X78064	Hoste et al. [47]
Sheep	UK	JF680986	Unpublished
Roe deer	Russia: Ryazan region	KR020010-KR020012	Unpublished
Goat; cattle	Iran: Khuzestan Province	JF276024-JF276026	Ghasemikhah et al. [27]
Sheep	Iran: Ardebil Province, Meshkin Shahr district	KJ755061	Unpublished
Sheep	Iran: Ardebil Province, Meshkin Shahr district	KF880745	Unpublished
Human	Iran: Guilan Province, Langroud district	KF872228	Unpublished
Human	Iran: Guilan Province, Fouman district	KY355024; KY355027	Sharifdini et al. [32]
Goat; sheep	Iran: Kohgiluyeh and Boyerahmad Province	MK275235-MK275238	Present study

## Discussion

In the present study, the only *Trichostrongylus* species isolated from different parts of one of the southwestern Iranian provinces was *T. vitrinus*. Sixteen ITS2 genotypes were found among the studied isolates, all of the nucleotide changes were SNPs and most in the 5'-end of the fragment. Comparison of the present isolates with those from other parts of Iran and other countries revealed two main clades. Isolates from Iran, the UK, New Zealand, Australia and Russia were distributed in one main clade and South African samples comprised another clade.

Previous studies in Iran has detected other *Trichostrongylus* spp. in livestock. In Isfahan Province (central Iran), *T. axei*, *T. vitrinus* and *T. colubriformis* were isolated from sheep [38] and *T. vitrinus*, *T. capricola*, *T. probolorus*, *T. skrjabini*, *T. axei* and *T. colubriformis* from sheep and goats [39]. In Khuzestan Province (West Iran), *T. vitrinus*, *T. colubriformis* [40] and in Golestan Province in North Iran, *T. axei* were isolated from goats and sheep [41].

Human infections with several *Trichostrongylus* spp. have also been reported in Iran, including *T. lerouxi* from northern, central, and southwestern regions [42], *T. orientalis*, *T. colubriformis*, and *T. axei*, *T. vitrinus*, *T. capricola*, *T. probolurus* and *T. skrjabini* in Isfahan (central), *T. orientalis*, *T. colubriformis*, *T. axei*, and *T. vitrines *in Khuzestan Province (West) and also along the Caspian Sea in North Iran [43]. Recent studies from northern provinces showed *T. colubriformis*, *T. vitrinus*, *T. longispicularis* and *T. axei* in Guilan Province [32] and *T. colubriformis*, and *T. axei* in Mazandaran Province [44]. Also, in East Azerbaijan in northwest Iran, *T. probolurus*, *T. colubriformis* and *T. vitrinus*, have been reported.

*Trichostrongylus vitrinus* has broad distribution within Iran and is the dominant species isolated from 95% of sheep breeding farms in England, Scotland, Wales and Northern Ireland [8]. The widespread distribution of *T. vitrinus* may be related to its ability to withstand a wide range of environmental conditions; moreover, Blackburn et al. [45] showed that *T. vitrinus* larvae can grow in temperatures < 8 °C and this is one distinguishing feature compared to other *Trichostrongylus* species.

Although, we sampled in three parts of Kohgiloyeh and Boyerahmad Province, finding only *T. vitrinus* in our study remains unclear. This contrasts with multiple species found in other Iranian studies with samples usually obtained from a single region or slaughterhouse in a province. Amongst the myriad environmental- (temperature, humidity, microclimate, season and type of vegetation), host (age, host movement, anthelmintic treatment, immunity and genetic resistance), and parasite-related factors (larval survival) [3], drug resistance is a possible explanation. There is only one Iranian, drug resistance study from Khuzestan Province the neighboring region of Kohgiloyeh and Boyerahmad Province and this showed a higher levamisole drug resistance of *T. vitrinus* compared to other *Trichostrongylus* species in sheep flocks [40]. A recent study in the UK also showed *T. vitrinus* as the sole *Trichostrongylus* species with monepantel resistance in a sheep/cattle farm [46]. Another study revealed higher anti-helminthic resistance and higher prevalence of *T. vitrinus* in treated livestock in comparison to other species including *T. colibriformis* and *T. axei* in Scotland [9]. These results contrast to the findings in New Zealand that demonstrated greater resistance to albendazole/levamizole in *T. colubriformis* compared to *T. vitrinus* and *T. axei* [4]. Veterinary authorities in Kohgiloyeh and Boyerahmad Province report an increasing use of anti-parasitic drugs by nomadic, rural and industrial herders (M. Sadghi, personal communication) that may reinforce the hypothesis of drug resistance in *T. vitrinus* isolates in studied areas.

Our study included a relatively high number of *T. vitrinus* isolates in comparison to other studies, resulting to detecting several haplotypes in a number of different phylogenetic positions; 16 (1 to 16) and 4 (A to D) haplotypes were found in the long and short size fragments of ITS2 sequences of *T. vitrinus*, respectively. The long nucleotide fragment of the ITS2 gene was associated with eight nucleotide changes between haplotypes and five of eight polymorphic points (37, 51, 62, 96 and 147 nucleotides) were observed more frequently in several haplotypes. The DNA chromatograph showed > 1 peak (concomitant existence of ≥ two nucleotides) at these points in some isolates in the forward and reverse sequences, although the nucleotide chromatograph was dominant for one nucleotide in each position. The alignment of our sequences with those from GenBank showed that these nucleotide positions were also highly polymorphic in a number of isolates. These highly variable points seem not to be the same in the different ITS copies even in one isolate. These findings are consistent with those of Hoste et al. [47] who reported these SNPs in the ITS2 copies of *T. vitrinus*, which account for intra-individual variation.

The topology of phylogenetic tree of all isolates showed two distinct clades, A and B, with clade A containing more haplotypes and countries than clade B (South Africa only). All north-northwest and some southwest (haplotypes A and B) and western isolates of Iran as well as isolates from Ryazan region of Russia, Australia [47], New Zealand [48] and the UK [49] had identical phylogenetic position in clade A. Haplotypes C and D from southwest Iran and two haplotypes from New Zealand [48] and one from the UK formed a subclade within clade A. Similarly, Ghasemikhah et al*.* [27] showed by Bayesian inference that *T. vitrinus* from Khuzestan Province (JF2760224) was in a sub cluster with *T. vitrinus* from the UK (AY439027). The link between the UK, Australia, New Zealand and Iran could be the export of livestock from the UK during colonial times. In the 19th and 20th centuries, British forces were stationed in some parts of Iran and the British Oil Company, established in 1909, was headquartered in Khuzestan [50]. Moreover, distribution of haplotypes of Kohgiloyeh and Boyerahmad Province in two positions in the clade A is consistent with higher genetic flow due to the movement of livestock, notably sheep and goats, by nomadic tribes who travel to and from cold to warmer regions during different seasons of the year [51] and exposure to strains of *Trichostrongylus* from various foci in a confined time interval. The similarity of isolates from Russian and the northern provinces of Iran is due to close geographic proximity and presence of Russian troops before and after both World Wars in North Iran.

## Conclusions

*Trichostrongylus vitrinus* was the only species isolated from sheep and goat in Kohgiloyeh and Boyerahmad Province southwest Iran where higher drug resistance may contribute in the dominance of this species. The similarity of Iranian, UK, New Zealand and Australian isolates may be explained by colonial activity of the UK and notable relationship with these countries in previous centuries. High genetic variations of southwest Iran may be due to annual movement of livestock by nomad’s activities and exposure of livestock with different strains of *T. vitrinus* in different regions and higher genetic flow between these isolates. To our knowledge, this is the first comprehensive study to evaluate the intraspecific population structure of *T. vitrinus* isolates from different foci of Iran and other countries. More research is needed to characterize further the molecular epidemiology of *Trichostrongylus* in Iran and examine important factors like treatment practice and the possible role of drug pressure and drug resistance on the predominance of certain isolates in different regions. Moreover, understanding better the transmission between livestock and man could lead to better control strategies.

## Data Availability

Not applicable.

## Abbreviations

ITS2: Internal transcribed spacer 2
ITS2-rDNA: Internal transcribed spacer 2 of ribosomal DNA
PCR-RFLP: PCR-restriction fragment length polymorphism
RAPD: Randomized amplified polymorphism DNA
PCR-SSCP: PCR-single-strand conformational polymorphism
PCR-DGGE: PCR-denaturing gradient gel electrophoresis
K-B: Kohgiloyeh and Boyerahmad
SNPs: Single nucleotide polymorphisms
STs: Genotypes
